# Neuroprotective Effects of Methyl Caffeate against Hydrogen Peroxide-Induced Cell Damage: Involvement of Caspase 3 and Cathepsin D Inhibition

**DOI:** 10.3390/biom10111530

**Published:** 2020-11-09

**Authors:** Danuta Jantas, Jakub Chwastek, Janusz Malarz, Anna Stojakowska, Władysław Lasoń

**Affiliations:** 1 Department of Experimental Neuroendocrinology, Maj Institute of Pharmacology, Polish Academy of Sciences, Smętna 12, 31-343 Kraków, Poland; chwastek@if-pan.krakow.pl (J.C.); lason@if-pan.krakow.pl (W.L.); 2 Department of Phytochemistry, Maj Institute of Pharmacology, Polish Academy of Sciences, Smętna 12, 31-343 Kraków, Poland; malarzj@if-pan.krakow.pl (J.M.); stoja@if-pan.krakow.pl (A.S.)

**Keywords:** 6-OHDA, glutamate, doxorubicin, staurosporine, pepstatin A, calpain inhibitor, SH-SY5Y cells, primary neuronal cell cultures

## Abstract

Finding effective neuroprotective strategies to combat various neurodegenerative disorders still remain a clinically unmet need. Methyl caffeate (MC), a naturally occurring ester of caffeic acid, possesses antioxidant and anti-inflammatory activities; however, its role in neuroprotection is less investigated. In order to better characterize neuroprotective properties of MC, we tested its effectiveness in various models of neuronal cell injury in human neuroblastoma SH-SY5Y cells and in mouse primary neuronal cell cultures. MC at micromolar concentrations attenuated neuronal cell damage induced by hydrogen peroxide (H_2_O_2_) in undifferentiated and neuronal differentiated SH-SY5Y cells as well as in primary cortical neurons. This effect was associated with inhibition of both caspase-3 and cathepsin D but without involvement of the PI3-K/Akt pathway. MC was neuroprotective when given before and during but not after the induction of cell damage by H_2_O_2_. Moreover, MC was protective against 6-OHDA-evoked neurotoxicity in neuronal differentiated SH-SY5Y cells via inhibition of necrotic and apoptotic processes. On the other hand, MC was ineffective in models of excitotoxicity (induced by glutamate or oxygen–glucose deprivation) and even moderately augmented cytotoxic effects of the classical apoptotic inducer, staurosporine. Finally, in undifferentiated neuroblastoma cells MC at higher concentrations (above 50 microM) induced cell death and when combined with the chemotherapeutic agent, doxorubicin, it increased the cell damaging effects of the latter compound. Thus, neuroprotective properties of MC appear to be limited to certain models of neurotoxicity and depend on its concentrations and time of administration.

## 1. Introduction

Hydroxycinnamic acids (HCAs) belong to the family of phenolic compounds which are widespread in nature, and among them, caffeic (CA), ferulic, p-coumaric and sinapic acids are most abundant [1,2,3]. They are synthesized by plants as secondary metabolites in various forms, e.g., glycosides, amides, or esters, and play a chemopreventive role against various microorganisms. Although phenolic acids are available in everyday human diet as they are commonly found in grains, nuts, oilseeds, fruits, vegetables, and beverages, their bioavailability is rather limited [1,2,4]. Thus, much effort has been devoted to finding HCA-rich plant species for efficient extraction and isolation of active compounds. In addition, various synthetic derivatives (esters, amides) have been synthesized based on particular HCA-scaffolds [5,6,7,8,9,10,11]. They are supposed to display a higher activity than natural compounds although their biosafety should be closely monitored to limit potential side effects. Many studies demonstrated that HCAs and their derivatives possess robust antioxidant and anti-inflammatory activities under various experimental settings, thus their potential role in prevention and therapy of various pathologies, such as cancer, cardiovascular disorders, diabetes, and neurodegenerative diseases has been proposed [1,2,12]. According to the literature published so far CA and its derivatives seem to be the most promising candidates for neuroprotective agents since they possess relatively higher antioxidant activity and are protective against a wider range of harmful factors when compared to other HCAs [8,10,12,13]. The representatives of this class of phenolic compounds most often studied so far in pre-clinical models of neurodegeneration include caffeic acid phenyl ester (CAPE, the main active component of propolis), chlorogenic acid (CGA, present in abundance in green coffee), chicoric acid, CA and rosmarinic acid (active component of rosemary herb) [14,15,16,17,18,19]. These compounds are characterized by pleiotropic mechanisms of action encompassing the activation of Nrf2/ARE/HO-1 pathway and induction of phase II enzymes (SOD, GSH, NADPH oxidase, xanthine oxidase), inhibition of calpains, attenuation of apoptotic and neuroinflammatory processes, inhibition of p38 and JNK pathways, induction of autophagy, activation of pro-survival PI3-K/Akt and MAPK/ERK1/2 pathways and stimulation of production of growth factors (NGF, BDNF, GDNF, VEGF) [5,6,7,15,18,20,21,22,23,24,25]. 

Methyl caffeate (MC) is a naturally occurring ester of CA which can be found in numerous plant taxa, including the Asteraceae family members with a worldwide distribution, like Lactuca spp. [26,27]. A number of reports on the biological activities of MC have focused on its potent anti-cancer properties [28,29,30,31,32]. Additionally, anti-microbial, anti-inflammatory, anti-platelet, anti-diabetic, hepatoprotective, or anti-senescence properties of MC have also been shown [33,34,35,36,37,38]. However, the neuroprotective properties of MC are less investigated. Garrido et al. [8] has shown that only esters of CA, including also MC, but not CA, ferulic acid or ferulate esters were able to attenuate the hydrogen peroxide (H_2_O_2_)-induced cell damage in rat PC12 neuronal-like cells. Moreover, in the model of serum deprived PC12 cells, of all tested various HCAs and their esters only CA and its esters (including also MC) have been shown to promote cell survival and to enhance the NGF-induced neurite outgrowth in PC12 cells [5]. It could be hypothesized that MC could be an efficient neuroprotective compound due to its wide distribution in plants and increased lipophilicity when compared to CA [8,10,12].

Thus, in order to better characterize the MC-mediated neuroprotection, we tested effectiveness of this CA derivative against various types of neuronal cell injury in human neuroblastoma SH-SY5Y cells, a widely accepted neuronal cellular model for Parkinson’s disease (PD) [39] and in mouse primary cortical neuronal cell cultures. Since previous data showed that another CA derivative, 3,5-DCQA (3,5-dicaffeoylquinic acid) protected SH-SY5Y cells against H_2_O_2_-induced cell damage [40], we used this compound for comparative studies with MC. 3,5-DCQA has been reported to be also present in large amounts in the Asteraceae family [26]. Both CA derivatives were isolated from two species of the genus Lactuca L. (Asteraceae, tribe Cichorieae) at the Department of Phytochemistry of the Maj Institute of Pharmacology, Polish Academy of Sciences. We employed models of neuronal cell damage evoked by the oxidative stress inducers (H_2_O_2_ and 6-hydroxydopamine)-induced, apoptotic agents (staurosporine, doxorubicin) or excitotoxic factors (glutamate; oxygen glucose deprivation, OGD). To further investigate the mechanisms of neuroprotection mediated by MC, we measured the activity of various proteases (caspase-3, cathepsin D) which have been reported to be activated after H_2_O_2_ exposure in our previous studies [41,42] and we verified the involvement of various signaling pathways by using inhibitors of calpain, cathepsin D or PI3-K/Akt pathway. 

## 2. Materials and Methods 

### 2.1. Chemicals

Dulbecco’s Modified Eagle’s Medium (DMEM), Neurobasal A, supplement B27 (w/o antioxidants), fetal bovine serum (FBS) and FluoroBrite™ DMEM were from Gibco (Invitrogen, Paisley, UK). The Cytotoxicity Detection Kit was from Roche Diagnostic (Mannheim, Germany). Caspase-3 (Ac-DEVD-AMC) and cathepsin D (MOCA-Gly-Lys-Pro-Ile-Leu-Phe-Phe-Arg-Leu-Lys(Dnp)-D-Arg-NH2) fluorogenic substrates were obtained from Enzo Life Sciences (New York, NY, USA). All other reagents were from Sigma (Sigma-Aldrich Chemie GmbH, Velbert, Germany).

### 2.2. Caffeic Acid Derivatives

**Figure 1:**
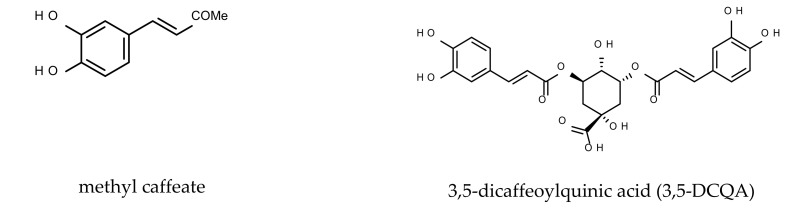
Chemical structure of methyl caffeate (MC) and 3,5-dicaffeoylquinic acid (3,5-DCQA).

Methyl caffeate (MC; methyl (E)-3-(3,4-dihydroxyphenyl)prop-2-enoate) and 3,5-DCQA were provided by Department of Phytochemistry of the Maj Institute of Pharmacology, Polish Academy of Sciences. MC was isolated from a methanolic extract of the dried Lactuca aculeata Boiss. et Kotschy callus tissue (efficiency 0.14 mg/g dry weight) as described previously [27]. 3,5-DCQA was obtained in a substantial amount (8.35 mg/g dry weight) from a butanolic fraction of a hydroalcoholic extract from the dried hairy roots of Lactuca virosa L. according to the protocol described previously [26]. The purity of both isolated phenolic compounds was > 90% as revealed by HPLC. For additional mechanistic studies, MC was purchased from Abcam (ab142321) with >98% purity according to the product data sheet. We did not notice any differences in the protective response of MC between both providers. The chemical structures of the studied two CA derivatives are shown in Figure 1.

### 2.3. SH-SY5Y Cell Culture 

The human SH-SY5Y neuroblastoma cells (ATCC, passages 5–20) were grown in DMEM supplemented with 10% heat-inactivated FBS and 100 units/mL of penicillin and 100 μg/mL of streptomycin as described previously [41]. The cells were maintained at 37 °C in an atmosphere with saturated humidity containing 95% air and 5% CO_2_. After reaching 80% confluence, cells were counted with a LUNA^TM^ Automatic Cell Counter (Logos Biosystems, Inc., Korea) and seeded at a density of 5 × 10^4^ and 2.5 × 10^5^ cells per well into 96- and 24-well plates, respectively. To obtain differentiated cells (RA-SH-SY5Y), the cells were plated at a half of the densities mentioned above and cultured in medium supplemented with retinoic acid (RA, 10 µM) for 6 days, during which the culture medium was changed every two days. One day before treatment, the culture medium for undifferentiated (UN-SH-SY5Y) and RA-SH-SY5Y cells was replaced with DMEM containing antibiotics and 1% FBS.

### 2.4. Primary Neuronal Cell Cultures

Cortical neuronal cell cultures were prepared from mouse C57Bl/6J embryos (15/16 day of gestation) and were cultured as described previously [43]. The protocol for generating the primary neuronal cell cultures was in accordance with local and international guidelines on the ethical use of animals. Animal care followed official governmental guidelines and all efforts were made to minimize the number of animals used and their suffering. The neuronal cells were suspended and maintained in Neurobasal A medium supplemented with B27 (without antioxidants) and antibiotics (0.06 μg/mL penicillin and 0.1 μg/mL streptomycin). The cells were seeded at a density of 6 × 10^4^ and 2.5 × 10^5^ cells per well in 96- and 24-well plates, respectively. Before cell seeding, the plates were covered overnight with poly-L-ornithine (0.05 mg/mL). The cells were cultured at 37 °C in a humidified atmosphere containing 5% CO_2_ for 7 days prior to experimentation with medium exchange every 2 days. 

### 2.5. Cell Treatment

First, SH-SY5Y cells or primary neurons were treated with MC or 3,5-DCQA at concentrations 1–100 μM and 1–50 μM, respectively, for 24 h to assess biosafety of the compounds. In the next series of experiments, the cells were pre-treated for 30 min. with MC or 3,5-DCQA (0.1–50 μM) followed by 24 h exposure to H_2_O_2_ (0.25 mM, 0.5 mM, and 0.2 mM for UN-, RA-SH-SY5Y and primary neurons, respectively). Additionally, we tested effectiveness of MC under co-treatment (given together with H_2_O_2_)- and post-treatment (given 30 min after H_2_O_2_) schedules. Moreover, we examined the effect of MC and 3,5-DCQA under 30 min pre-treatment schedule against doxorubicin (Dox; 0.375 and 1 μM for UN- and RA-SH-SY5Y cells, respectively)- or staurosporine (St; 0.25 μM in UN-SH-SY5Y cells)-induced cell damage in SH-SY5Y cells and against glutamate (Glu, 1 mM) or staurosporine (St, 0.5 μM)-evoked cell death in primary neurons. Finally, we studied the effect of MC in the model of oxygen glucose deprivation (OGD)-induced cell damage in primary neurons and in the model of SH-SY5Y cell damage induced by 6-hydroxydopamine (6-OHDA; 100 and 200 μM for UN- and RA-SH-SY5Y cells). The antioxidant N-acetylcysteine (NAC, 1 mM) was used as a positive control for oxidative stress-induced cell damage models (H_2_O_2_ or 6-OHDA) [41,42], whereas the NMDA receptor antagonist, MK-801 (1 μM) was employed for glutamate- or OGD-evoked neuronal demise [43,44]. The effective concentrations of particular cell damaging factors (H_2_O_2_, Dox, Glu, St, 6-OHDA) and time of treatment (24 h; OGD 3 h followed by 24 h of reperfusion) were established in our previous studies, in which these factors reduced cell viability by approximately 50% [41,42,43,44,45]. For mechanistic studies, inhibitors of cathepsin D (pepstatin A; PsA; 0.3 μM), calpain (MDL28170; 20 μM) and PI3-K, LY294002 (10 μM) were employed.

MC and 3,5-DCQA stock solutions (10 mM) were prepared in 70% ethanol which was aliquoted and kept frozen at −20 °C. The final solutions of phenolic compounds were prepared freshly in distilled water. Ac-DEVD-CHO (10 mM), MDL28170 (10 mM), LY294002 (10 mM), PsA (10 mM), and St (50 mM) stock solutions were prepared in DMSO, and Dox (5 mM) in distilled water. The final solutions of the tested chemicals were prepared in distilled water. The H_2_O_2_ (25 and 50 mM) stock solutions were prepared freshly from stabilized 30% hydrogen peroxide diluted in distilled water. The 6-OHDA (10 mM) and Glu (100 mM) stock solutions were prepared immediately before use in distilled water and 100 mM NaOH, respectively. The liquids were prepared and procedure for OGD was done in accordance with previous study [46]. All agents were added to the culture medium at the indicated concentrations under light-limited conditions. Each experimental set of the control cultures was supplemented with the appropriate vehicles, and the solvent was present in cultures at a final concentration of 0.1%.

### 2.6. Cell Viability Assay

Cell viability was quantified using a tetrazolium salt colorimetric assay with 3-[4,5-dimethylthiazol-2-yl]-2,5-diphenyltetrazolium bromide (MTT) as described previously [42]. The data were normalized to the vehicle-treated cells (100%) and expressed as a percentage of the control ± SEM established from 3–9 independent experiments with 3–5 replicates.

### 2.7. LDH Release Assay

The level of lactate dehydrogenase (LDH) released into culture media after 24 h of particular treatments was measured with Cytotoxicity detection kit (Roche) as described previously [42]. The data were normalized to the vehicle-treated cells and expressed as a percent of the control ± SEM from 3–9 independent experiments with 3–5 replicates.

### 2.8. Propidium Iodide Staining and Flow Cytometry

To confirm the results obtained by biochemical cell viability/toxicity assays, the UN- and RA-SH-SY5Y cells were cultured in 24-well plate format and after 24 h of treatment with particular agents were stained with propidium iodide (PI) according to the method described previously [42]. 1 × 10^4^ cells were analyzed using a BD FACS Canto II System and BD FACSDiva™ v5.0.1 Software (BD Biosciences, San Jose, CA, USA) in the fluorescence channel for PerCP-Cy5-5-A (red fluorescence). The cells exhibiting loss of cell membrane integrity (PI positive) represent necrotic and late apoptotic cells. Data are presented as a percentage of PI positive cells (± SEM) established from 3 independent experiments with 2 replicates.

### 2.9. CalceinAM/Hoechst 33,342 Live Imaging and MAP-2/Hoechst 33,342 Immunofluorescence

In order to confirm biochemical cell viability data at the morphological level, primary neuronal cell cultures growing in 24-well plate format after 24 h of treatment with MC (1-50 μM) and H_2_O_2_ (0.2 mM) were live imaged by using double staining with CalceinAM (marker of viable cells) and Hoechst 33,342 (nuclear marker) dyes or after fixation in 4% paraformaldehyde were immunostained with neuronal marker (mouse anti-MAP-2, 1:250, M9942, Sigma-Aldrich), as described previously [42,43]. For live cell imaging, the cells after labeling were placed in FluoroBrite™ DMEM whereas for immunocytochemistry the samples after staining were mounted in ProLong®Gold antifade reagent (Invitrogen, Waltham, MA, USA). The samples were evaluated by using an inverted fluorescence microscope (AxioObserver, Carl Zeiss, Jena, Germany) with an excitation wavelength of 480 nm (CalceinAM, Alexa®488) and 355 nm (Hoechst 33342) equipped with a black-white camera (Axio-CamMRm, Carl Zeiss). Four microphotographs for each panel (480 or 355 nm) were taken for each tested group in duplicates from 2 independent experiments. The numbers of pyknotic (condensed and/or fragmented) and healthy nuclei were counted semi-manually from a 355-nm panel using AxioVison software as described previously [42].

### 2.10. Caspase-3 Activity Assay

The caspase-3 activity in UN- or RA-SH-SY5Y cells growing in 6-well format and treated for 9 h with H_2_O_2_ and MC or for 24 h with 6-OHDA (200 μM) and MC (10 μM) was measured using the fluorogenic substrate Ac-DEVD-AMC (50 μM) as described in detail previously [42]. The caspase-3 inhibitor, Ac-DEVD-CHO (20 μM) was used to verify the assay specificity. The data (expressed as mean relative fluorescence units, RFU) first were normalized to protein level (measured by the BCA method) and next calculated as a percent of vehicle-treated cells and presented as the mean ± SEM from 3 separate experiments with 2 repetitions each.

### 2.11. Cathepsin D Activity Assay

Cathepsin D activity in UN- and RA-SH-SY5Y cells treated for 18 h with H_2_O_2_ and MC was measured using a fluorometric method employing the fluorogenic substrate AMC-Gly-Lys-Pro-Ile-Leu-Phe-Phe-Arg-Leu-Lys(Dnp)-D-Arg-NH2 as described previously [42]. PsA (0.3 μM) was used as a positive control for the assay. Cathepsin D activity expressed in RFU first was calculated per mg of protein and next normalized to the vehicle-treated cells. The results are shown as the mean± SEM from 3-4 independent experiments with 2 replicates.

### 2.12. Statistical Analysis

Data were analyzed using the Statistica software (StatSoft Inc., Tulsa, OK, USA). The analysis of variance (one-way ANOVA) and post-hoc Tukey’s test for multiple comparisons were used to show statistical significance with assumed *p* < 0.05.

## 3. Results

### 3.1. The Effects of MC and 3,5-DCQA on H_2_O_2_-Induced Cell Damage in UN- and RA-SH-SY5Y Cells

**Figure 2:**
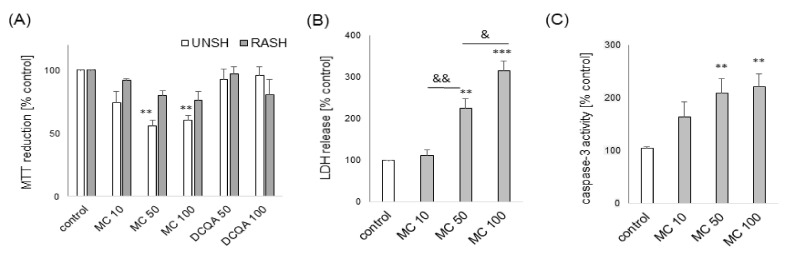
(**A**) The effect of MC (10–100 μM) or 3,5-DCQA (50 and 100 μM) on cell viability of undifferentiated (UN-) and retinoic acid-differentiated (RA-) SH-SY5Y cells after 24 h of treatment (measured with MTT reduction assay). (**B**) The cytotoxic effect of MC (10–100 μM) in UN-SH-SY5Y cells after 24 h of treatment as measured with LDH release assay. (**C**) The effect of MC (10–100 μM) on caspase-3 activity in UN-SH-SY5Y cells after 9 h of treatment. Data were normalized to vehicle-treated cells and are presented as the mean ± SEM. *** *p* *<* 0.001 and ** *p* *<* 0.01 vs. vehicle-treated cells; ^&&^ *p* *<* 0.01 and ^&^ *p* *<* 0.05 a higher vs. lower concentration of MC.

Twenty four hours of treatment with 3,5-DCQA at concentrations up to 100 μM did not evoke any detrimental effect on UN- or RA-SH-SY5Y cells as confirmed by cell viability assay (Figure 2A). MC caused no cell damage in both UN- and RA-SH-SY5Y cells up to 10 μM but at concentrations of 50 and 100 μM it reduced cell viability by about 40% in UN- but not in RA-SH-SY5Y cells (Figure 2A). This detrimental effect at higher concentrations of MC in undifferentiated cells was connected with its cytotoxic and pro-apoptotic properties as confirmed by LDH release (Figure 2B) and caspase-3 activity (Figure 2C) assays, respectively. 

**Figure 3:**
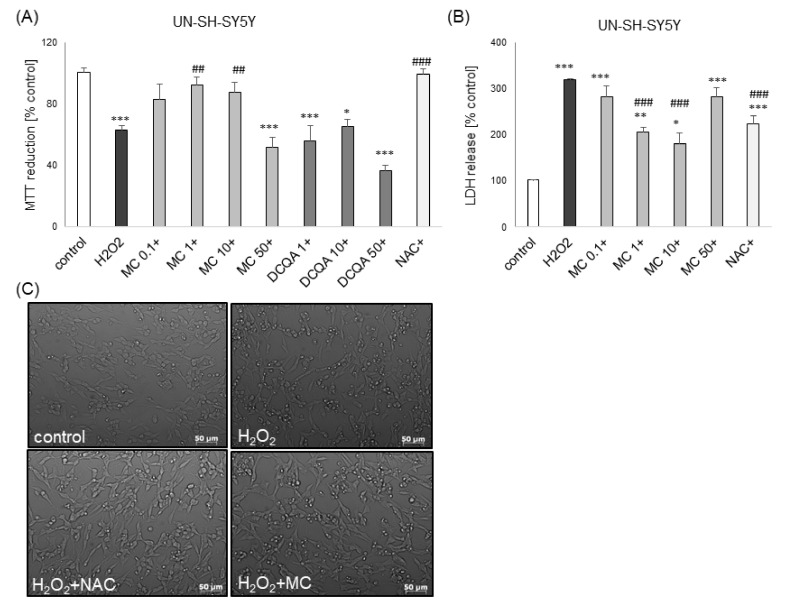
The protective effects of methyl caffeate (MC) against hydrogen peroxide (H_2_O_2_)-evoked UN-SH-SY5Y cell damage. (**A**,**B**) Cell viability (**A**) and toxicity (**B**) in UN-SH-SY5Y cells pre-treated for 30 min. with MC (0.1-50 μM) or 3,5-DCQA (1-50 μM) or co-treated with N-acetylcysteine (NAC, 1 mM) followed by 24 of treatment with H_2_O_2_ (0.25 mM) measured by MTT reduction and LDH release assays, respectively. Data were normalized to the vehicle-treated cells and are presented as the mean ± SEM. *** *p* *<* 0.001, ** *p* *<* 0.01 and * *p* *<* 0.05 vs. vehicle-treated cells; ^###^ *p* *<* 0.001 and ^##^ *p* *<* 0.01 vs. H_2_O_2_-treated cells. (**C**) Representative DIC (differential interference contrast) images of UN-SH-SY5Y cells treated for 24 h with MC (10 μM) or N-acetylcysteine (1 mM) and H_2_O_2_ (0.25 mM).

**Figure 4:**
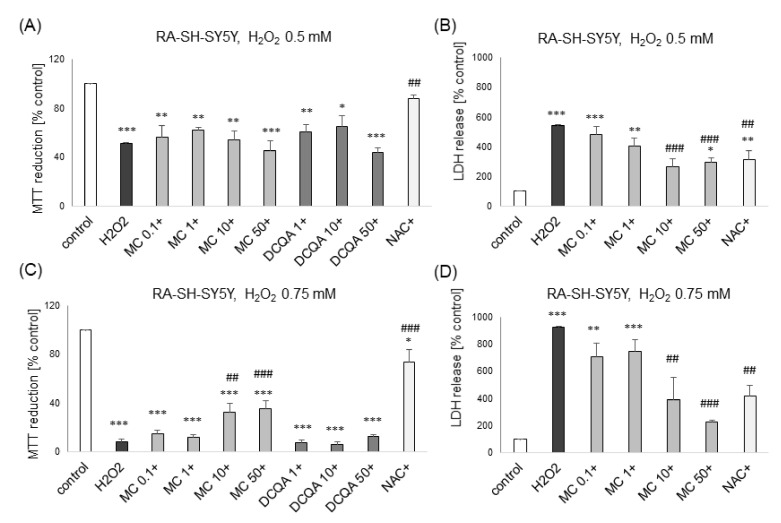
The protective effects of MC against hydrogen peroxide (H_2_O_2_)-evoked RA-SH-SY5Y cell damage. (**A**,**C**) Cell viability of RA-SH-SY5Y cells pre-treated for 30 min. with MC (0.1–50 μM) or 3,5-DCQA (1–50 μM) or co-treated with N-acetylcysteine (NAC, 1 mM) followed by 24 of treatment with 0.5 mM (**A**) or 0.75 mM (**C**) H_2_O_2_ measured by MTT reduction assay. (**B**,**D**) Cell toxicity of RA-SH-SY5Y cells pre-treated for 30 min. with MC (0.1–50 μM) or co-treated with N-acetylcysteine (NAC, 1 mM) followed by 24 h of treatment with H_2_O_2_ with 0.5 mM (**A**) or 0.75 mM (**C**) H_2_O_2_ as measured by LDH release assay. Data were normalized to the vehicle-treated cells and are presented as the mean ± SEM. *** *p* < 0.001, ** *p* < 0.01, and * *p* < 0.05 vs. vehicle-treated cells; ^###^ *p* < 0.001 and ^##^ *p* < 0.01 vs. H_2_O_2_-treated cells.

Of the two tested caffeic acid derivatives at wide range of concentrations (0.1–50 μM), only MC showed neuroprotective effects. This compound attenuated the H_2_O_2_-induced cell damage at concentrations of 1 and 10 μM, and 10 and 50 μM in UN-SH-SY5Y and RA-SH-SY5Y cells, respectively, as evidenced by the MTT reduction test (Figure 3A and Figure 4C) and LDH release assay (Figure 3B and Figure 4B,D). In UN-SH-SY5Y cells that effect was at similar level as the protection mediated by the antioxidant N-acetyl-cysteine (NAC, 1 mM) (Figure 3A,B), whereas in RA-SH-SY5Y the prevention was only partial (Figure 4C). Moreover, in RA-SH-SY5Y cells we did not find any attenuating effect of MC on the H_2_O_2_-evoked reduction in cell viability when cells were moderately damaged (H_2_O_2_ 0.5 mM; ca. 50% injury) (Figure 4A) but we observed neuroprotective effects when more severe damage occurred (H_2_O_2_ 0.75 mM; ca. 80% injury) (Figure 4C).

**Figure 5:**
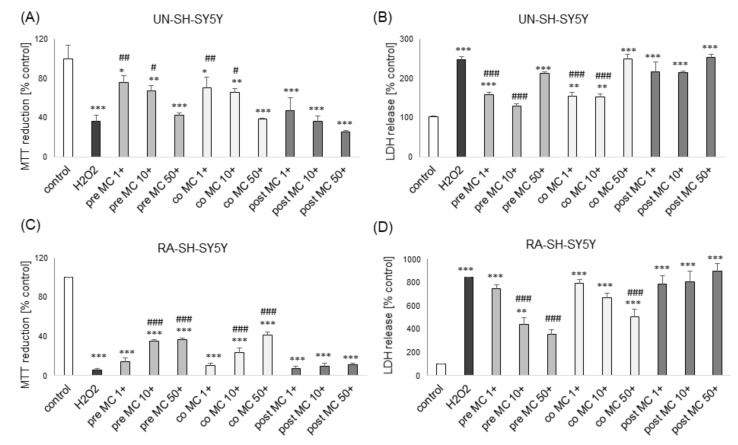
The protective effects of MC against hydrogen peroxide (H_2_O_2_)-evoked UN- or RA-SH-SY5Y cell damage are retained under pre- and co-treatment but not in post-treatment conditions. (**A**,**C**) Cell viability of UN-(A) and RA-(C) SH-SY5Y cells pre-treated (30 min. before H_2_O_2_), co-treated and post-treated (30 min. after H_2_O_2_) with MC (1–50 μM) followed by 24 of treatment with H_2_O_2_ (0.25 and 0.5 mM for UN- and RA-SH-SY5Y cells, respectively) as measured by MTT reduction assay. (**B**,**D**) Cell toxicity in UN-(B) and RA-(D) SH-SY5Y cells pre-treated, co- and post-treated with MC (1–50 μM) followed by 24 of treatment with H_2_O_2_ (0.25 and 0.5 mM for UN- and RA-SH-SY5Y cells, respectively) as measured by LDH release assay. Data were normalized to the vehicle-treated cells and are presented as the mean ± SEM. *** *p* < 0.001, ** *p* < 0.01 and * *p* < 0.05 vs. vehicle-treated cells; ^###^ *p* < 0.001, ^##^ *p* < 0.01 and ^#^ *p* < 0.05 and vs. H_2_O_2_-treated cells.

However, in the LDH release assay we found a significant reduction of H_2_O_2_-induced toxicity by MC (10 and 50 μM) in models of moderate and high cell damage (Figure 4B,D). Additionally, we found that MC protected both cell phenotypes of SH-SY5Y cells when given 30 min before (pre-) and during (co-) exposure to H_2_O_2_ but it was not effective when given 30 min after (post-) the cell damaging factor as evidenced by the MTT reduction test (Figure 5A,C) and LDH release assay (Figure 5B,D). The neuroprotective effects of MC (10 μM) and NAC against H_2_O_2_-evoked cell damage in UN-H-SY5Y cells were confirmed morphologically using DIC (differential interference contrast) light microscopy (Figure 3C). 

### 3.2. The MC-Mediated Protection against H_2_O_2_-Induced Cell Damage in SH-SY5Y Cells is Connected with Inhibition of Apoptotic and Necrotic Processes

**Figure 6:**
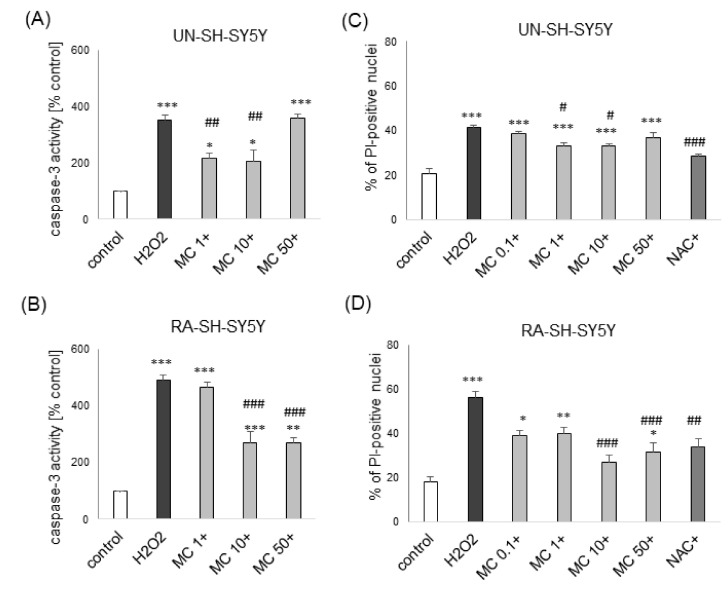
The protective effects of MC against hydrogen peroxide (H_2_O_2_)-evoked UN- or RA-SH-SY5Y cell damage are mediated by inhibition of apoptotic and necrotic processes. (**A**,**B**) The effect of 30-min pre-treatment with MC (1–50 μM) followed by 9 h of exposure to H_2_O_2_ (0.25 and 0.75 mM for UN- and RA-SH-SY5Y cells, respectively) on caspase-3 activity in UN-(A) and RA-(B) SH-SY5Y cells. Data were normalized to vehicle-treated cells and are presented as the mean ± SEM. (**C**,**D**) The effect of 30-min pre-treatment with MC (0.1–50 μM) or co-treated with N-acetylcysteine (NAC, 1 mM) followed by 24 h of exposure to H_2_O_2_ (0.25 and 0.75 mM for UN- and RA-SH-SY5Y cells, respectively) on the number of necrotic nuclei measured by propidium ioide (PI) staining and flow cytometry in UN-(C) and RA-(D) SH-SY5Y cells. Data are presented as the mean ± SEM percentage of necrotic nuclei. *** *p* < 0.001, ** *p* < 0.01 and * *p* < 0.05 vs. vehicle-treated cells; ^###^ *p* < 0.001, ^##^ *p* < 0.01 and ^#^ *p* < 0.05 vs. H_2_O_2_ -treated cells.

Since our previous data showed an involvement of apoptotic and necrotic processes in H_2_O_2_ induced cell damage in SH-SY5Y cells [41,42], we tested if MC could influence these processes. We demonstrated a significant induction of caspase-3 activity after 9 h of exposure of SH-SY5Y cells to H_2_O_2_ (0.25 and 0.75 mM for UN- and RA-SH-SY5Y cells, respectively) which was significantly attenuated by a caspase 3 inhibitor (data not shown) and MC caffeate at concentrations 1–10 μM and 10–50 μM for UN- and RA-SH-SY5Y cells, respectively (Figure 6A,B). We also found a significant MC-induced reduction (at 1–10 and 10–50 μM for UN- and RA-SH-SY5Y cells, respectively) of the H_2_O_2_ -evoked increase in the number of necrotic (PI-positive) nuclei (Figure 6C,D).

### 3.3. The Mechanisms of MC-Mediated Protection against H_2_O_2_-Induced Cell Damage in SH-SY5Y Cells Involve Inhibition of Cathepsin D but not Activation of PI3-K/Akt Signaling

**Figure 7:**
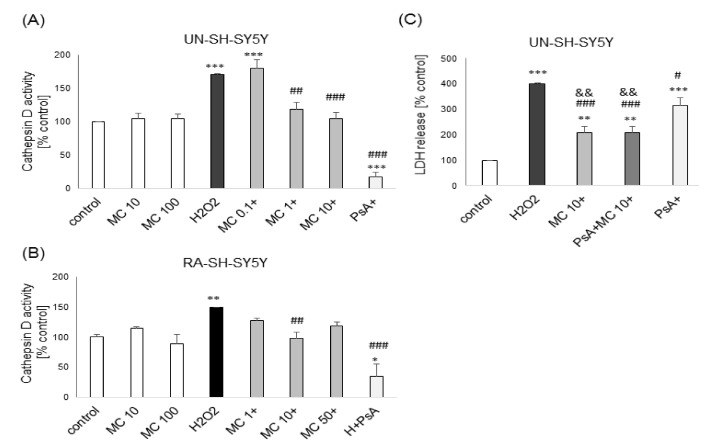
The protective effects of MC against hydrogen peroxide (H_2_O_2_)-evoked UN- or RA-SH-SY5Y cell damage are mediated by inhibition of cathepsin D. (**A**,**B**) The effect of 30-min pre-treatment with MC (0.1–50 μM) followed by 18 h exposure to H_2_O_2_ (0.25 and 0.75 mM for UN- and RA-SH-SY5Y cells, respectively) on cathepsin D activity in UN- (A) and RA- (B) SH-SY5Y cells. Cathepsin D inhibitor, pepstatin A (PsA, 0.3 μM) was used as a positive control for the assay. (**C**) The effect of cathepsin D inhibitor on the methyl caffeate-mediated protection against the H_2_O_2_-induced cell damage in UN-SH-SY5Y cells. The cells were pre-treated for 30 min with cathepsin D inhibitor, pepstatin A (PsA, 0.3 μM) and next treated with MC (10 μM) 30 min before H_2_O_2_ (0.25 mM) exposure. After 24 h of treatment, cytotoxicity was assessed by LDH release assay. Data were normalized to vehicle-treated cells and are presented as the mean ± SEM. *** *p* < 0.001, ** *p* < 0.01 and * *p* < 0.05 vs. vehicle-treated cells; ^###^ *p* < 0.001, ^##^ *p* < 0.01 and ^#^ *p* < 0.05 vs. H_2_O_2_-treated cells; ^&&^ *p* < 0.01 vs. H_2_O_2_+PsA-treated group.

In the present study, we observed an increase in cathepsin D activity after 18 h of exposure of UN- and RA-SH-SY5Y cells to H_2_O_2_ which confirms our previous results [41,42]. This effect was significantly attenuated by MC (1–10 and 10 μM for UN- and RA-SH-SY5Y cells, respectively) and cathepsin D inhibitor, PsA (0.3 μM) (Figure 7A,B). Additionally, by using cytotoxicity (LDH release) assay we demonstrated that protection mediated by MC was significantly higher than PsA-mediated but was not changed after combined treatment with both neuroprotective compounds (Figure 7C). 

Since previous reports demonstrated an involvement of inhibition of calpains and activation of PI3-K/Akt intracellular pathway in neuroprotection mediated by caffeic acid- and its derivatives [6,22,23], we verified these mechanisms in our study by using calpain (MDL28170) and PI3-K (LY294002) inhibitors. We showed that cytoprotection mediated by MC was more robust than that mediated by MDL28170 (20 μM) and has not been changed after combined treatment with both compounds (Table 1). Moreover, we found that LY294002 (10 μM) did not inhibit the protective effect of MC (10 μM) against the H_2_O_2_-evoked UN-SH-SY5Y cell damage (Table 2). 

### 3.4. MC is Protective against 6-OHDA-Induced Cell Damage in SH-SY5Y Cells: The Impact of Cell Differentiation State

**Figure 8:**
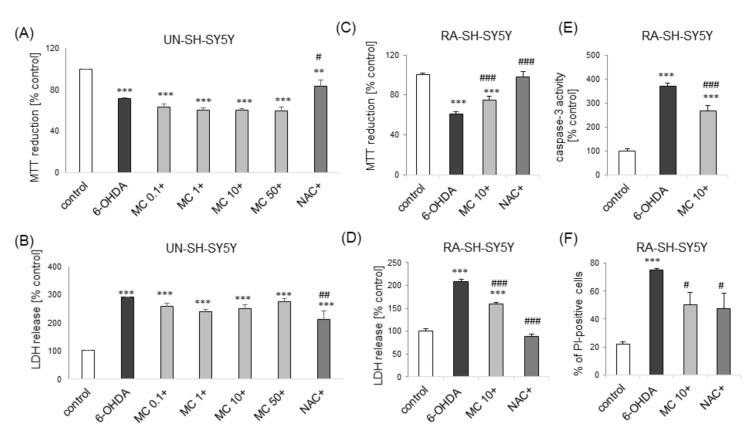
The effect of MC on 6-OHDA-induced cell damage in UN- and RA-SH-SY5Y cells. (**A**,**B**) Cell viability (**A**) and toxicity (**B**) in UN-SH-SY5Y cells pre-treated for 30 min. with MC (0.1–50 μM) or co-treated with N-acetylcysteine (NAC, 1 mM) followed by 24 h of treatment with 6-OHDA (100 μM) as measured by MTT reduction (**A**) and LDH release (**B**) assays. Data were normalized to vehicle-treated cells and are presented as the mean ± SEM. (**C**,**D**) Cell viability (**C**) and toxicity (**D**) in RA-SH-SY5Y cells pre-treated for 30 min. with MC (10 μM) or co-treated with N-acetylcysteine (NAC, 1 mM) followed by 24 h of treatment with 6-OHDA (200 μM) as measured by MTT reduction (**C**) and LDH release (**D**) assays. Data were normalized to vehicle-treated cells and are presented as the mean ± SEM. (**E**) The effect of 30-min pre-treatment with MC (10 μM) followed by 24 h of exposure to 6-OHDA (200 μM) in RA-SH-SY5Y cells on caspase-3 activity. Data were normalized to vehicle-treated cells and are presented as the mean ± SEM. (**F**) The effect of 30-min pre-treatment with MC (10 μM) or co-treated with N-acetylcysteine (NAC, 1 mM) followed by 24 h of exposure to 6-OHDA (200 μM) in RA-SH-SY5Y cells on the number of necrotic nuclei measured by propidium ioide (PI) staining. Data are presented as the mean ± SEM percentage of necrotic nuclei. *** *p* < 0.001 and ** *p* < 0.01 vs. vehicle-treated cells; ^###^ *p* < 0.001, ^##^ *p* < 0.01 and ^#^ *p* < 0.05 vs. 6-OHDA-treated cells.

Next, we tested the effect of pre-treatment with MC against SH-SY5Y cell damage induced by dopaminergic neurotoxin, 6-OHDA in UN- and RA-SH-SY5Y cells. We observed protection afforded by NAC (1 mM) but not by MC (0.1-50 μM) against 6-OHDA (100 μM)-induced cell damage in UN-SH-SY5Y cells (Figure 8A,B). On the contrary, we found that MC (10 μM) partially attenuated the cell damage induced by 6-OHDA (200 μM) in RA-SH-SY5Y cells as confirmed by cell viability test (Figure 8C) and LDH release assay (Figure 8D). Moreover, this protection was connected with attenuation of apoptotic (caspase-3 activity; Figure 8E) and necrotic (PI-positive cells; Figure 8F) processes.

### 3.5. The Effects of MC against Dox- and St-Induced Cell Damage in SH-SY5Y Cells

We tested the effects of MC in cell damage model evoked by the chemotherapeutic drug doxorubicin (Dox) in UN- and RA-SH-SY5Y cells. No protection was observed after treatment with this caffeic acid derivative (0.1–50 μM) in both cell phenotypes (Figure S1). However, in UN-SH-SY5Y cells we observed a significant decrease (by about 14–18%) in cell viability evoked by MC (1–50 μM) when compared to Dox-treated cells (Figure S1A) as confirmed by the MTT reduction test but at the LDH release level such synergism has not been noticed (Figure S1B). In the model of staurosporine (St)-induced cell damage of UN-SH-SY5Y, we observed ca. 24% reduction in cell viability after exposure to this toxin (Figure S1E), which was not accompanied by an increase in cytotoxicity (Figure S1F). However, MC at a concentration of 50 μM significantly increased cell damage induced by St which was confirmed by MTT reduction (Figure S1E) and LDH release (Figure S1F) assays. In the LDH assay, also 10 μM of MC increased cell death in the St-treated cells (Figure S1F). 

### 3.6. The Effects of MC and 3,5-DCQA against Various Types of Neuronal Cell Injury in Primary Neuronal Cell Cultures

**Figure 9:**
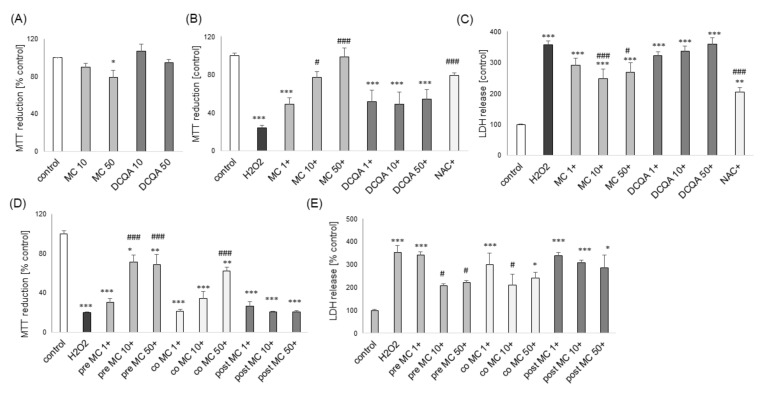
The effect of MC on H_2_O_2_-induced cell damage in primary neuronal cell cultures. (**A**) The effect of MC (10 and 50 μM) or 3,5-DCQA (10 and 50 μM) on cell viability in primary neuronal cell cultures after 24 h of treatment as measured with MTT reduction assay. (**B**,**C**) Cell viability (B) and toxicity (**C**) in primary neurons pre-treated for 30 min. with MC (1–50 μM) or 3,5-DCQA (1–50 μM), or co-treated with N-acetylcysteine (NAC, 1 mM) followed by 24 h of treatment with H_2_O_2_ (0.2 mM) as measured by MTT reduction (**B**) and LDH release (**C**) assays. (**D**,**E**) Cell viability (**D**) and toxicity (**E**) in primary neurons pre-treated (30 min. before H_2_O_2_), co-treated and post-treated (30 min. after H_2_O_2_) with MC (1-50 μM) followed by 24 of treatment with H_2_O_2_ (0.2 mM) as measured by MTT reduction (**D**) and LDH release (**E**) assays. Data were normalized to vehicle-treated cells and are presented as the mean ± SEM. *** *p* < 0.001, ** *p* < 0.01 and * *p* < 0.05 vs. vehicle-treated cells; ^###^ *p* < 0.001 and ^#^ *p* < 0.05 vs. H_2_O_2_-treated cells.

**Figure 10:**
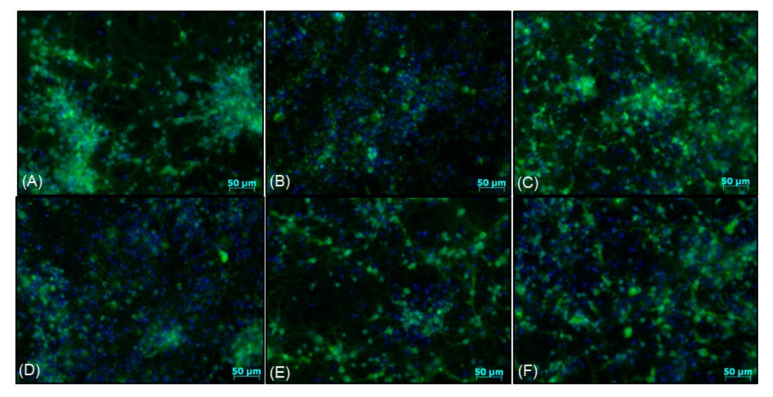
Representative microphotographs of primary neurons pre-treated for 30 min. with MC (1–50 μM) or co-treated with N-acetylcysteine (NAC, 1 mM) followed by 24 h of exposure to H_2_O_2_ (0.2 mM). After the treatment the cells were double-stained with CalceinAM (green)/Hoechst 33,342 (blue) dyes and live imaged with fluorescence microscopy. (**A**—control); (**B**)—H_2_O_2_; (**C**)—H_2_O_2_+NAC; (**D**)—H_2_O_2_+MC 1; (**E**)—H_2_O_2_+MC 10; (**F**)—H_2_O_2_+MC 50.

**Figure 11:**
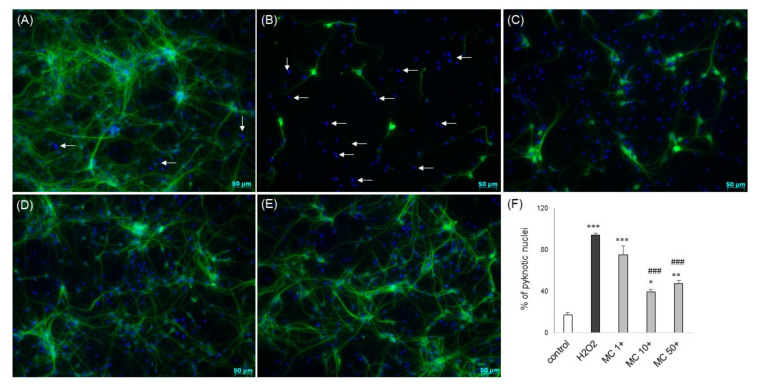
(**A**–**E**) Representative microphotographs of primary neurons pre-treated for 30 min. with MC (1–50 μM) followed by 24 h of exposure to H_2_O_2_ (0.2 mM). After the treatment the cells were fixed and immunostained with a neuronal marker (anti-MAP-2, green) and contrastained with nuclear dye Hoechst 33,342 (blue; examples of pyknotic nuclei are indicated by arrows). (**A**)—control; (**B**)—H_2_O_2_; (**C**)—H_2_O_2_+MC 1; (**D**)—H_2_O_2_+MC 10; (**E**)—H_2_O_2_+MC 50. (**F**) Histogram showing an estimation of the number of pyknotic nuclei from Hoechst 33,342 staining. Nuclei showing bright blue florescence (condensed or fragmented) were semi-manually counted and presented as the mean percentage of pyknotic nuclei/all nuclei ± SEM. *** *p* < 0.001, ** *p* < 0.01 and * *p* < 0.05 vs. vehicle-treated cells; ^###^ *p* < 0.001 vs. H_2_O_2_-treated cells.

Twenty four hours of treatment with 3,5-DCQA at concentrations up to 50 μM did not evoke any detrimental effect in primary neurons but MC at a concentration of 50 μM reduced cell viability by about 20% (Figure 9A). MC but not 3,5-DCQA at concentrations of 10 an 50 μM attenuated the H_2_O_2_-induced cell damage in primary neuronal cell cultures as evidenced by the MTT reduction test (Figure 9B) and LDH release assay (Figure 9C). This effect was similar to the protection mediated by antioxidant N-acetyl-cysteine (NAC, 1 mM) (Figure 9B,C). Like in SH-SY5Y cells, MC protected primary neurons when given 30 min before (pre-) and during (co-) exposure to H_2_O_2_ but it was not effective when given 30 min after (post-) cell damaging factor (Figure 9D,E). The neuroprotective effects of MC against the H_2_O_2_-evoked cell damage were confirmed also at morphological level using live cell imaging (CalceinAM/Hoechst 33342) (Figure 10) or anti-MAP-2 immunostaining (Figure 11). Moreover, MC (10 and 50 μM) attenuated the H_2_O_2_-induced increase in the number of pyknotic (apoptotic and necrotic) nuclei as evaluated by Hoechst 33,342 staining (Figure 11F). 

We did not observe any protection mediated by MC (1–50 μM) against neuronal cell damage evoked by the pro-apoptotic factor, staurosporine (St, 0.5 μM) (Figure S2A,B). However, MC at a concentration of 50 μM and 10-50 μM increased cell damage induced by St as found in MTT reduction (Figure S2A) and LDH (Figure S2B) assays, respectively. MC (1-50 μM) also did not change neuronal cell death induced by the excitotoxic factor, glutamate (1 mM) (Figure S2C,D) or oxygen–glucose deprivation (OGD, Figure S2E,F) in 7 DIV primary neuronal cell cultures, however, the NMDA antagonist, MK-801 (1 μM) was neuroprotective in these models.

## 4. Discussion

In this study, we showed that of the two tested CA derivatives only MC revealed significant protective effects against oxidative stress–induced neuronal cell damage as evidenced by various biochemical and morphological tests. The concentration-dependent beneficial effect of MC on cell survival upon H_2_O_2_ exposure was evidenced here for the first time in differentiated and undifferentiated neuroblastoma SH-SY5Y cells as well as in primary neuronal cell cultures. The comparable data obtained in both human- and rodent cell-based models strengthen the position of SH-SY5Y cells as a reliable first screening platform for neuroprotective drugs reducing the animal use for these purposes [39,46,47]. Our data suggest the ability of MC at micromolar concentrations (1–50 μM) to reduce oxidative stress-induced cell damage. In the previous study in rat PC12 cells, it was demonstrated that CA and its esters (methyl, ethyl, propyl and butyl) showed similar free radical scavenging activities (IC50~15 μM) in cell-free assays which were better than Trolox C or ferulic acid and its esters. However, among the tested agents only CA esters (concentrations 5 and 25 μM) including also MC have been found to be cytoprotective [8]. The above mentioned facts suggest that other than antioxidant mechanisms could be responsible for the neuroprotective effects of CA esters. The cell survival promoting effects of CA and its derivatives could be also cell type dependent, since in the study mentioned above, CA did not affect the H_2_O_2_-evoked cell damage in dopaminergic PC12 cells [8] but it was protective (50 μM) in a similar model of cell injury in primary cerebellar granule cells [13]. However, in the latter study CGA, ferulic acid or quinic acids were inefficient [13]. It should be mentioned that in our study we observed beneficial effects of MC in various neuronal cell phenotypes: dopaminergic (SH-SY5Y) [39,46] and mixture of glutamatergic/GABAergic (primary cortical neurons) cells [48] which rather suggests universal mechanisms of MC-mediated neuroprotection at least in the H_2_O_2_-induced model of cell damage. It should be noted that MC was earlier reported to be protective in non-neuronal cells as shown in the oxidative stress-induced hepatic damage in rats but in that study also other HCAs (CGA, CA, ferulic acid) were effective [38]. 

It was previously suggested that the increased biological activity of MC could be a result of increased lipophilicity which could be achieved by esterification [8,10,12]. At cellular level, it means an increased intracellular uptake of MC, and it is highly probable that such phenomena could explain the neuroprotective effects of MC but not 3,5-DCQA, observed in our study (Table 3). Our data did not confirm previous results found in UN-SH-SY5Y where 2 h of pretreatment with 3,5-DCQA or 3,4-DCQA (10–50 μM) partially attenuated the H_2_O_2_-induced cell damage [40]. There are several potential factors which could be responsible for the discrepancies between Kim’s [40] and our study, like different number of cell passages, various cell densities during testing, different serum level in culture medium during experiments (10 vs. 1% FBS, respectively), various time of pretreatment (2 h vs. 30 min, respectively) or different concentration of H_2_O_2_ used (0.1 vs. 0.25 mM, respectively) to mention a few. In addition, another cell type, cardiomyocyte H9C2 cell line, 24 h pre-treatment with 3,5-DCQA (3–20 μM) produced the protective effect against oxidative stress-induced cell damage induced by tertbutyl hydroperoxide [49]. However, we did not notice any protection offered by 3,5-DCQA against oxidative stress-induced cell damage not only in UN-SH-SY5Y or RA-SH-SY5Y cells but also in primary neurons. Moreover, 3,5-DCQA was also ineffective in apoptotic (St, Dox) and excitotoxic (Glu) models of neuronal cell damage (Table 3). Another observation from the present study was that MC protected SH-SY5Y cells or primary neurons against H_2_O_2_-induced cell damage when added to cell culture before (30 min. pre-treatment) or at the same time (co-treatment) as the damaging agent, but was quite inefficient when given at the later time (30 min. post-treatment). One could suggest that this phenomenon is connected with direct reaction of MC with H_2_O_2_ and this may explain the lack of protective effect when it was added 30 min after H_2_O_2_ treatment. However, we excluded that mechanism on the basis hypothesis that the number of hydroxyl groups could determinate the ROS scavenging activity of CA derivatives [10]. In our case MC contains two hydroxyl groups whereas 3,5-DCQA six, but only the former has been found to be protective. We rather think that when MC was given 30 min after H_2_O_2_, the cellular damage reached an advanced stage, at which MC was not protective any longer. It should be noted that most previous studies testing neuroprotective potential of CA derivatives usually used pre-treatment protocols (24 h, 2 h, or 1 h before toxin exposure) but without any comparisons between them [13,19,24,40]. The relatively narrow time window of MC efficacy (the best effects in pre-treatment group, slightly worsened in co-treatment group and lack of effect in post-treatment group) found in our study suggests that it might be mainly recommended for preventive strategy of neuronal damage, but not for reducing already existing brain injury, e.g., in the late phase of stroke. This conclusion is also supported by our data from models of excitotoxicity (glutamate- or OGD-evoked neuronal damage) where MC was not protective in contrast to the NMDA receptor antagonist, MK-801. These findings distinguish MC from other CA derivatives since previous studies showed neuroprotective effects of CA or its derivatives (CGA, CAPE, or rosmarinic acid) against glutamate-evoked neuronal cell death [13,19,50,51] as well as in animal brain ischemia models [51,52,53]. 

In contrast to CA or its most often studied derivatives (CGA, CAPE, or rosmarinic acid) [16,18,19], no data on intracellular mechanisms involved in neuroprotection mediated by MC were available. Previously, we showed that the mechanisms of H_2_O_2_-induced cell damage involved activation of both caspase-3 and lysosomal protease, cathepsin D [41,42]. In the present work we confirmed these data and demonstrated for the first time that MC significantly inhibited both H_2_O_2_-induced caspase-3 and cathepsin D activities in neuronal-like cells (SH-SY5Y cells). These results suggest involvement of inhibition of apoptotic and necrotic processes in the neuroprotective action of MC. It should be noted that previous studies demonstrated that CA or its derivatives inhibited apoptotic processes in neuronal cells which were induced by various factors (e.g., low potassium level, H_2_O_2_, serum deprivation) [5,8,13,19,40] and our present results extend these data by showing the effects of MC in the oxidative stress-based model. It is also in line with previous data from non-neuronal cells (kidney epithelium cell line LLC-PK) where MC (100 μM) reduced the contrast-induced caspase-3 activity and increased cell survival [54]. However, the MC effects in apoptotic models could be inducer-specific since in our study we not only did not find any protection offered by this compound against neuronal cell damage induced by staurosporine, an activator of caspase-3 dependent apoptosis [55], but we even observed an increased cytotoxicity after concomitant treatment with both agents in UN-SH-SY5Y and in primary neurons. Interestingly, we showed the ability of MC to inhibit the H_2_O_2_-induced cathepsin D which is mechanistically linked with the lysosomal cell death pathway [56]. Moreover, by comparison of protective effects of MC with effectiveness of the cathepsin D inhibitor, PsA, where the latter was significantly less protective than MC and there was no potentiation of the protection after concomitant treatment with both agents, we concluded that the inhibition of cathepsin D could be only partially responsible for the MC-mediated neuroprotection. To our knowledge, so far there is only one study showing the inhibitory effect of CA on lysosomal enzymes (including also cathepsin D) which was associated with its protective effects in the model of myocardial infarction in rats [57]. However, we cannot exclude that inhibition of other proteases, calpains could also play some role in the MC-mediated neuroprotection since in our previous work we observed their increased activity after H_2_O_2_ exposure [41]. Moreover, in the present study we found that the calpain inhibitor, MDL28170 protected cells against oxidative stress-induced cell damage to a smaller extent than MC and the protection range was not changed after administration of both compounds which suggests possible involvement of calpain inhibition in the MC-mediated neuroprotection. There are conclusive data showing the participation of activation of the pro-survival PI3-K/Akt pathway in the neuroprotective effects of CA and/or its derivatives [5,6,7,22]. However, in our study the inhibitor of PI3-K, LY294002 failed to block the neuroprotective effects of MC in the H_2_O_2_ model of cell damage which excludes the participation of this signaling pathway in the MC-mediated neuroprotection at least in this model of neuronal injury. Nevertheless, a previous docking study indicated that PI3-K was a potential molecular target for some esters of caffeate, which could explain their neurotrophic capacities in serum-deprived PC12 cells [5]. 

An additional new finding from the present study is the observation that MC attenuated, though to a smaller extent and in the RA-SH-SY5Y cells only, the cell damage induced by 6-OHDA, the neurotoxin widely used in animal models of Parkinson’s disease [58]. This effect was connected with inhibition of apoptotic and necrotic processes induced by 6-OHDA which is in line with our finding from the model of H_2_O_2_-induced cell damage. However, in contrast to the latter procedure, in the model of 6-OHDA-evoked cell death we did not observe any protection induced by MC in undifferentiated cells. This phenomenon is hard to explain at present and it needs further research. Usually, opposite effects have been found in cellular neuroprotection studies employing SH-SY5Y cells where differentiation process could mask the potential protection by the tested compounds [45,59]. Nevertheless, our results revealed some neuroprotective potential of MC against 6-OHDA-induced injury in dopaminergic cell phenotype thus this CA derivative could be considered as a potential therapeutic candidate for the prevention of neurodegeneration in PD. It should be noted that among CA derivatives, CAPE and PACA (N-propargyl caffeate amide) have been the most frequently studied so far in respect to their potential use in PD. For example, CAPE was protective against 6-OHDA-induced cell damage in SH-SY5Y cells [24,60] or in primary neuronal cell cultures [61] as well as in animal models of PD [62,63]. 

Another observation from our study is related to cytotoxic potential of MC at higher concentrations (50 and 100 μM) found in undifferentiated neuroblastoma cells which was connected with induction of apoptosis as confirmed by activation of caspase-3. This is in line with previous reports that have shown the anticancer properties of MC or CAPE in various cancer cell types, e.g. melanoma, lung carcinoma, breast cancer or glioblastoma [28,30,31,32]. Our observation that this compound alone at high concentration may decrease cell survival, confirms the view that phenolic compounds including CA possess both pro- and antioxidant properties [20]. Moreover, the augmenting effect of MC on Dox-induced reduction in cell viability in undifferentiated, but not in neuronally differentiated SH-SY5Y cells, found in our study, suggests that this compound can be considered as adjunctive drug in some forms of chemotherapy.

## 5. Conclusions

**Figure 12:**
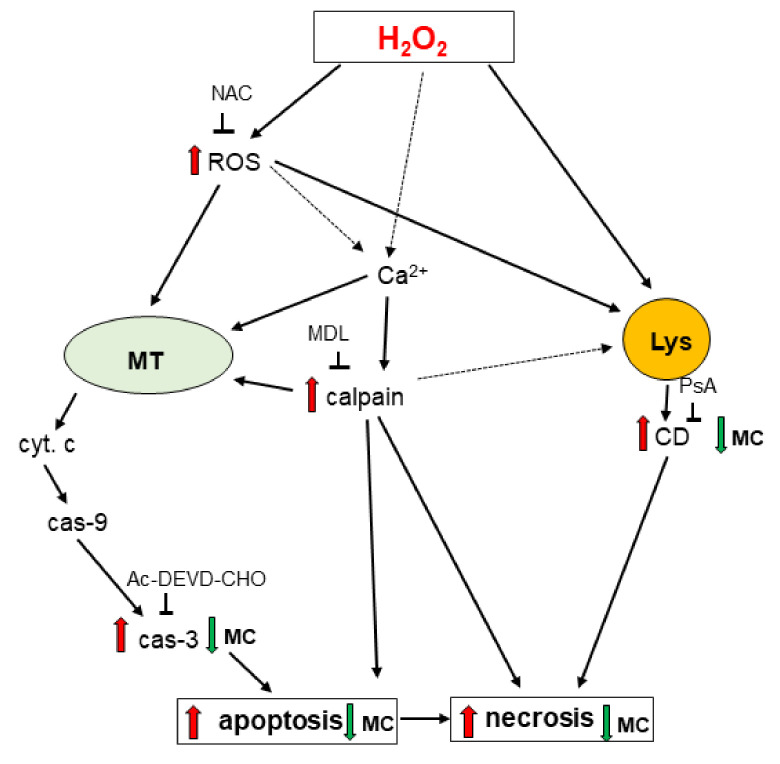
A schematic illustration of possible mechanisms by which methyl caffeate (MC) mediates neuroprotection against the H_2_O_2_-induced cell damage in undifferentiated (UN-) and retinoic acid (RA)-differentiated SHSY5Y cells. Cathepsin D inhibition and caspase-3 inhibition are proposed as candidate mechanisms associated with neuroprotection mediated by MC in oxidative stress (H_2_O_2_)-evoked neuronal cell damage. Ac-DEVD—Ac-DEVD-CHO, an inhibitor of caspase-3; cas-3—caspase-3; cas-9—caspase9; CD—cathepsin D; cyt.c—cytochrome c; H_2_O_2_—hydrogen peroxide; Lys—lysosome; MDL—MDL28170, an inhibitor of calpains; PsA—pepstatin A, an inhibitor of cathepsin D; ROS—reactive oxygen species.

In summary, the present study showed complex effects of MC in various in vitro models of neurotoxicity. This compound inhibited H_2_O_2_-induced neuronal cell damage and these effects were associated with inhibition of both caspase-3 and cathepsin D but without involvement of the PI3-K/Akt pathway (Figure 12). MC was neuroprotective when given before and during but not after induction of cell damage suggesting rather its utility as a preventive drug. Moreover, MC was protective against 6-OHDA-evoked neurotoxicity via inhibition of necrotic and apoptotic processes but that effect was limited to differentiated SH-SY5Y cells. On the other hand, MC was ineffective in models of excitotoxicity and even moderately augmented cytotoxic effects of staurosporine. Finally, in undifferentiated neuroblastoma cells MC at higher concentrations induced cell death and when combined with doxorubicin increased its cytostatic activity. Thus, neuroprotective properties of MC appear to be limited to some models of neurotoxicity and depend on its concentrations and time of its administration.

## Figures and Tables

**Table 1:** The effect of calpain inhibitor on the methyl caffeate-mediated protection against the hydrogen peroxide-induced cell damage in UN-SH-SY5Y cells.

	LDH Release [% control]
control	100.00 ± 0.01
H_2_O_2_	386.12 ± 3.67 ^***^
+MC	217.54 ± 19.59 ^***,###,&^
+MDL+MC	229.89 ± 21.57 ^***,###,&^	
+MDL	304.18 ± 27.84 ^***,#^

The cells were pre-treated for 30 min with the calpain inhibitor MDL28170 (MDL, 20 μM) and next treated with methyl caffeate (MC; 10 μM) 30 min before H_2_O_2_ (0.25 mM) exposure. After 24 h of treatment, cytotoxicity was assessed by LDH release assay. Data were normalized to the vehicle-treated cells and are presented as the mean ± SEM. *** *p* < 0.001 vs. vehicle-treated cells; ^###^ *p* < 0.001 and ^#^ *p* < 0.05 vs. H_2_O_2_-treated cells; ^&^ *p* < 0.05 vs. H_2_O_2_+MDL-treated group.

**Table 2:** The effect of PI3-K inhibitor (LY294002) on the methyl caffeate-mediated protection against the hydrogen peroxide-induced cell damage in UN-SH-SY5Y cells.

	LDH Release [% control]
control	100.18 ± 0.17
H_2_O_2_	386.69 ± 3.05 ^***^
+MC	201.81 ± 22.43 ^**,###^
+LY294+MC	245.54 ± 24.51 ^***,###^	
+LY294	427.73 ± 26.58 ^***^
LY294	131.95 ± 10.95

The cells were pre-treated for 30 min with PI3-K inhibitor LY294002 (LY294, 10 μM) and next treated with methyl caffeate (MC; 10 μM) 30 min before H_2_O_2_ (0.25 mM) exposure. After 24 h of treatment, cytotoxicity was assessed by LDH release assay. Data were normalized to the vehicle-treated cells and are presented as the mean ± SEM. *** *p* < 0.001, ** *p* < 0.01 vs. vehicle-treated cells; ^###^ *p* < 0.01 vs. H_2_O_2_-treated cells.

**Table 3:** Summary of MC and 3,5-DCQA effects in SH-SY5Y cells and in primary neurons in various models of neuronal cell damage.

Cell Type Cell Damage	UN-SH-SY5Y		RA-SH-SY5Y		Primary Neurons	
	MC	3,5-DCQA	MC	3,5-DCQA	MC	3,5-DCQA
H_2_O_2_	NP	0	NP	0	NP	0
6-OHDA	0	0 *	NP	n.s.	n.s.	n.s.
Staurosporine	D	0 *	n.s.	n.s.	D	0 *
Doxorubicin	D	0 *	0	0 *	n.s.	n.s.
Glutamate	n.s.	n.s.	n.s.	n.s.	0	0 *
OGD	n.s.	n.s.	n.s.	n.s.	0	n.s.

MC—methyl caffeate; NP—neuroprotection; 0—no effect; D—detrimental; n.s.—not studied; UN—undifferentiated cells; RA—retinoic acid-differentiated cells; OGD—oxygen–glucose deprivation; *—data not shown.

## Supplementary Materials

The following are available online at https://www.mdpi.com/2218-273X/10/11/1530/s1, Figure S1: The effect of MC on SH-SY5Y cell damage induced by doxorubicin and staurosporine. Figure S2: The effect of MC in primary neuronal cell damage induced by apoptotic (staurosporine) and excitotoxic (glutamate, oxygen glucose deprivation) factors.

## Abbreviations

3:5-DCQA—3,5-dicaffeoylquinic acid; 6-OHDA—6-hydroxydopamine; CA—caffeic acid; CAPE—caffeic acid phenyl ester; CGA—chlorogenic acid; Dox—doxorubicin; Glu—glutamate; HCAs—hydroxycinnamic acids; LDH—lactate dehydrogenase; MC—methyl caffeate; MTT—3-[4,5-dimethylthiazol-2-yl]-2,5-diphenyltetrazolium bromide; NAC—N-acetylcysteine; OGD—oxygen glucose deprivation; PI—propidium iodide; PsA—pepstatin A; ROS—reactive oxygen species; St- staurosporine.

## References

[1] Coman V., Vodnar D.C. (2019). Hydroxycinnamic acids and human health: Recent advances. J. Sci. Food Agric..

[2] El-Seedi H.R., El-Said A.M.A., Khalifa S.A.M., Göransson U., Bohlin L., Borg-Karlson A.-K., Verpoorte R. (2012). Biosynthesis, Natural Sources, Dietary Intake, Pharmacokinetic Properties, and Biological Activities of Hydroxycinnamic Acids. J. Agric. Food Chem..

[3] Kim Y.C. (2010). Neuroprotective phenolics in medicinal plants. Arch. Pharmacal Res..

[4] Manach C., Williamson G., Morand C., Scalbert A., Rémésy C. (2005). Bioavailability and bioefficacy of polyphenols in humans. I. Review of 97 bioavailability studies. Am. J. Clin. Nutr..

[5] Hosseini R., Moosavi F., Rajaian H., Silva T., E Silva D.M., Soares P., Saso L., Edraki N., Miri R., Borges F. (2016). Discovery of neurotrophic agents based on hydroxycinnamic acid scaffold. Chem. Biol. Drug Des..

[6] Hosseini R., Moosavi F., Silva T., Rajaian H., Hosseini S.Y., Bina S., Saso L., Miri R., Borges F., Firuzi O. (2018). Modulation of ERK1/2 and Akt Pathways Involved in the Neurotrophic Action of Caffeic Acid Alkyl Esters. Molecules.

[7] Moosavi F., Hosseini R., Rajaian H., Silva T., E Silva D.M., Saso L., Edraki N., Miri R., Borges F., Firuzi O. (2017). Derivatives of caffeic acid, a natural antioxidant, as the basis for the discovery of novel nonpeptidic neurotrophic agents. Bioorganic Med. Chem..

[8] Garrido J., Gaspar A., Garrido E.M., Miri R., Tavakkoli M., Pourali S., Saso L., Borges F., Firuzi O. (2012). Alkyl esters of hydroxycinnamic acids with improved antioxidant activity and lipophilicity protect PC12 cells against oxidative stress. Biochimie.

[9] Zhang X., He X., Chen Q., Lu J., Rapposelli S., Pi R. (2018). A review on the hybrids of hydroxycinnamic acid as multi-target-directed ligands against Alzheimer’s disease. Bioorganic Med. Chem..

[10] Razzaghi-Asl N., Garrido J., Khazraei H., Borges F., Firuzi O. (2013). Antioxidant Properties of Hydroxycinnamic Acids: A Review of Structure- Activity Relationships. Curr. Med. Chem..

[11] Białecka-Florjańczyk E., Fabiszewska A.U., Zieniuk B. (2019). Phenolic Acids Derivatives-Biotechnological Methods of Synthesis and Bioactivity. Curr. Pharm. Biotechnol..

[12] Shahidi F., Chandrasekara A. (2009). Hydroxycinnamates and their in vitro and in vivo antioxidant activities. Phytochem. Rev..

[13] Taram F., Winter A.N., Linseman D.A. (2016). Neuroprotection comparison of chlorogenic acid and its metabolites against mechanistically distinct cell death-inducing agents in cultured cerebellar granule neurons. Brain Res..

[14] Silva T., Oliveira C., Borges F. (2014). Caffeic acid derivatives, analogs and applications: A patent review (2009–2013). Expert Opin. Ther. Patents.

[15] Liu Q., Hu Y., Cao Y., Song G., Liu Z., Liu X. (2017). Chicoric Acid Ameliorates Lipopolysaccharide-Induced Oxidative Stress via Promoting the Keap1/Nrf2 Transcriptional Signaling Pathway in BV-2 Microglial Cells and Mouse Brain. J. Agric. Food Chem..

[16] Naveed M., Hejazi V., Abbas M., Kamboh A., Khan G.J., Shumzaid M., Ahmad F., Babazadeh D., FangFang X., Modarresi-Ghazani F. (2018). Chlorogenic acid (CGA): A pharmacological review and call for further research. Biomed. Pharm..

[17] Mikami Y., Kakizawa S., Yamazawa T. (2016). Essential Roles of Natural Products and Gaseous Mediators on Neuronal Cell Death or Survival. Int. J. Mol. Sci..

[18] Tolba M.F., Azab S.S., Khalifa A.E., Abdel-Rahman S.Z., Abdel-Naim A.B. (2013). Caffeic acid phenethyl ester, a promising component of propolis with a plethora of biological activities: A review on its anti-inflammatory, neuroprotective, hepatoprotective, and cardioprotective effects. IUBMB Life.

[19] Taram F., Ignowski E., Duval N., Linseman D.A. (2018). Neuroprotection Comparison of Rosmarinic Acid and Carnosic Acid in Primary Cultures of Cerebellar Granule Neurons. Molecules.

[20] Khan F.A., Maalik A., Murtaza G. (2016). Inhibitory mechanism against oxidative stress of caffeic acid. J. Food Drug Anal..

[21] Colonnello A., Aguilera-Portillo G., Rubio-López L.C., Robles-Bañuelos B., Rangel-López E., Cortez-Núñez S., Evaristo-Priego Y., Silva-Palacios A., Galván-Arzate S., García-Contreras R. (2020). Comparing the Neuroprotective Effects of Caffeic Acid in Rat Cortical Slices and Caenorhabditis elegans: Involvement of Nrf2 and SKN-1 Signaling Pathways. Neurotox. Res..

[22] Firuzi O., Moosavi F., Hosseini R., Saso L. (2015). Modulation of neurotrophic signaling pathways by polyphenols. Drug Des. Dev. Ther..

[23] Sul D., Kim H.-S., Lee D., Joo S.S., Hwang K.W., Park S.-Y. (2009). Protective effect of caffeic acid against beta-amyloid-induced neurotoxicity by the inhibition of calcium influx and tau phosphorylation. Life Sci..

[24] Tomiyama R., Takakura K., Takatou S., Le T.M., Nishiuchi T., Nakamura Y., Konishi T., Matsugo S., Hori O. (2017). 3,4-dihydroxybenzalacetone and caffeic acid phenethyl ester induce preconditioning ER stress and autophagy in SH-SY5Y cells. J. Cell. Physiol..

[25] Zaitone S.A., Ahmed E., Elsherbiny N.M., Mehanna E.T., El-Kherbetawy M.K., Elsayed M.H., Alshareef D.M., Moustafa Y.M. (2019). Caffeic acid improves locomotor activity and lessens inflammatory burden in a mouse model of rotenone-induced nigral neurodegeneration: Relevance to Parkinson’s disease therapy. Pharmacol. Rep..

[26] Stojakowska A., Malarz J., Szewczyk A., Kisiel W. (2011). Caffeic acid derivatives from a hairy root culture of Lactuca virosa. Acta Physiol. Plant..

[27] Stojakowska A., Malarz J. (2017). Bioactive phenolics from in vitro cultures of Lactuca aculeata Boiss. et Kotschy. Phytochem. Lett..

[28] Balachandran C., Emi N., Arun Y., Yamamoto Y., Ahilan B., Sangeetha B., Duraipandiyan V., Inaguma Y., Okamoto A., Ignacimuthu S. (2015). In vitro anticancer activity of methyl caffeate isolated from Solanum torvum Swartz. fruit. Chem. Interact..

[29] Bailly F., Toillon R.-A., Tomavo O., Jouy N., Hondermarck H., Cotelle P. (2013). Antiproliferative and apoptotic effects of the oxidative dimerization product of methyl caffeate on human breast cancer cells. Bioorganic Med. Chem. Lett..

[30] Khan R.S., Senthi M., Rao P.C., Basha A., Alvala M., Tummuri D., Masubuti H., Fujimoto Y., Begum A.S. (2014). Cytotoxic constituents of Abutilon indicum leaves against U87MG human glioblastoma cells. Nat. Prod. Res..

[31] Znati M., Ben Jannet H., Cazaux S., Souchard J.-P., Harzallah-Skhiri F., Bouajila J. (2014). Antioxidant, 5-Lipoxygenase Inhibitory and Cytotoxic Activities of Compounds Isolated from the Ferula lutea Flowers. Molecules.

[32] Fiuza S., Gomes C., Teixeira L., Da Cruz M.G., Cordeiro M., Milhazes N., Borges F., Marques M.P.M. (2004). Phenolic acid derivatives with potential anticancer properties––a structure–activity relationship study. Part 1: Methyl, propyl and octyl esters of caffeic and gallic acids. Bioorganic Med. Chem..

[33] Lim H., Park B.K., Shin S.Y., Kwon Y.S., Kim H.P. (2017). Methyl caffeate and some plant constituents inhibit age-related inflammation: Effects on senescence-associated secretory phenotype (SASP) formation. Arch. Pharmacal Res..

[34] Balachandran C., Duraipandiyan V., Al-Dhabi N.A., Balakrishna K., Kalia N.P., Rajput V.S., Khan I.A., Ignacimuthu S. (2012). Antimicrobial and Antimycobacterial Activities of Methyl Caffeate Isolated from Solanum torvum Swartz. Fruit. Indian J. Microbiol..

[35] Alson S.G., Jansen O., Cieckiewicz E., Rakotoarimanana H., Rafatro H., Degotte G., Francotte P., Frederich M. (2018). In-vitro and in-vivo antimalarial activity of caffeic acid and some of its derivatives. J. Pharm. Pharmacol..

[36] Gandhi G.R., Ignacimuthu S., Paulraj M.G., Sasikumar P. (2011). Antihyperglycemic activity and antidiabetic effect of methyl caffeate isolated from Solanum torvum Swartz. fruit in streptozotocin induced diabetic rats. Eur. J. Pharmacol..

[37] Pyo M.K., Lee Y., Yun-Choi H.S. (2002). Anti-platelet effect of the constituents isolated from the barks and fruits ofMagnolia obovata. Arch. Pharmacal Res..

[38] Bispo V.S., Dantas L.S., Filho A.B.C., Pinto I.F., Da Silva R.P., Otsuka F.A., Santos R.B., Santos A.C., Trindade D.J., Matos H.R. (2017). Reduction of the DNA damages, Hepatoprotective Effect and Antioxidant Potential of the Coconut Water, ascorbic and Caffeic Acids in Oxidative Stress Mediated by Ethanol. Anais da Academia Brasileira de Ciências.

[39] Lázaro D.F., Pavlou M.A.S., Outeiro T.F. (2017). Cellular models as tools for the study of the role of alpha-synuclein in Parkinson’s disease. Exp. Neurol..

[40] Kim S.-S., Park R.-Y., Jeon H.-J., Kwon Y.-S., Chun W. (2005). Neuroprotective effects of 3,5-dicaffeoylquinic acid on hydrogen peroxide-induced cell death in SH-SY5Y cells. Phytother. Res..

[41] Chwastek J., Jantas D., Lasoń W. (2017). The ATM kinase inhibitor KU-55933 provides neuroprotection against hydrogen peroxide-induced cell damage via a γH2AX/p-p53/caspase-3-independent mechanism: Inhibition of calpain and cathepsin D. Int. J. Biochem. Cell Biol..

[42] Jantas D., Chwastek J., Grygier B., Lasoń W. (2020). Neuroprotective Effects of Necrostatin-1 Against Oxidative Stress-Induced Cell Damage: An Involvement of Cathepsin D Inhibition. Neurotox. Res..

[43] Jantas D., Lech T., Gołda S., Pilc A., Lasoń W. (2018). New evidences for a role of mGluR7 in astrocyte survival: Possible implications for neuroprotection. Neuropharmacology.

[44] Domin H., Jantas D., Smiałowska M. (2015). Neuroprotective effects of the allosteric agonist of metabotropic glutamate receptor 7 AMN082 on oxygen-glucose deprivation- and kainate-induced neuronal cell death. Neurochem. Int..

[45] Jantas D., Greda A., Leskiewicz M., Grygier B., Pilc A., Lasoń W. (2015). Neuroprotective effects of mGluR II and III activators against staurosporine- and doxorubicin-induced cellular injury in SH-SY5Y cells: New evidence for a mechanism involving inhibition of AIF translocation. Neurochem. Int..

[46] Lopes F.M., Schröder R., Júnior M.L.C.D.F., Zanotto-Filho A., Müller C.B., Pires A.S., Meurer R.T., Colpo G.D., Gelain D.P., Kapczinski F. (2010). Comparison between proliferative and neuron-like SH-SY5Y cells as an in vitro model for Parkinson disease studies. Brain Res..

[47] Leist M. (2014). Consensus report on the future of animal-free systemic toxicity testing. ALTEX.

[48] De Groot M.W.G.D.M., Dingemans M.M.L., Rus K.H., De Groot A., Westerink R.H.S. (2013). Characterization of Calcium Responses and Electrical Activity in Differentiating Mouse Neural Progenitor Cells In Vitro. Toxicol. Sci..

[49] Bi Y.-M., Wu Y.-T., Chen L., Tan Z.-B., Fan H.-J., Xie L.-P., Zhang W.-T., Chen H.-M., Li J., Liu B. (2018). 3,5-Dicaffeoylquinic acid protects H9C2 cells against oxidative stress-induced apoptosis via activation of the PI3K/Akt signaling pathway. Food Nutr. Res..

[50] Rebai O., Belkhir M., Sánchez-Gómez M.V., Matute C., Fattouch S., Amri M. (2017). Differential Molecular Targets for Neuroprotective Effect of Chlorogenic Acid and its Related Compounds Against Glutamate Induced Excitotoxicity and Oxidative Stress in Rat Cortical Neurons. Neurochem. Res..

[51] Wei X., Zhao L., Ma Z., Holtzman D.M., Yan C., Dodel R., Hampel H., Oertel W., Farlow M.R., Du Y. (2004). Caffeic acid phenethyl ester prevents neonatal hypoxic-ischaemic brain injury. Brain.

[52] Kim I.H., Yan B.C., Park J.H., Yeun G.H., Yim Y., Ahn J.H., Lee J.-C., Hwang I.K., Cho J.H., Kim Y.-M. (2013). Neuroprotection of a Novel Synthetic Caffeic Acid-Syringic Acid Hybrid Compound against Experimentally Induced Transient Cerebral Ischemic Damage. Planta Medica.

[53] Liang G., Shi B., Luo W., Yang J. (2015). The protective effect of caffeic acid on global cerebral ischemia-reperfusion injury in rats. Behav. Brain Funct..

[54] Kim K.O., Lee D., Hiep N.T., Song J.H., Lee H.-J., Lee D., Kang K.S. (2019). Protective Effect of Phenolic Compounds Isolated from Mugwort (Artemisia argyi) against Contrast-Induced Apoptosis in Kidney Epithelium Cell Line LLC-PK1. Molecules.

[55] Koh J.-Y., Wie M.B., Gwag B.J., Sensi S.L., Canzoniero L.M.T., Demaro J., Csernansky C., Choi D.W. (1995). Staurosporine-Induced Neuronal Apoptosis. Exp. Neurol..

[56] Castino R., Bellio N., Nicotra G., Follo C., Trincheri N.F., Isidoro C. (2007). Cathepsin D–Bax death pathway in oxidative stressed neuroblastoma cells. Free. Radic. Biol. Med..

[57] Kumaran K.S., Prince P.S.M. (2010). Preventive effect of caffeic acid on lysosomal dysfunction in isoproterenol-induced myocardial infarcted rats. J. Biochem. Mol. Toxicol..

[58] Airavaara M., Parkkinen I., Konovalova J., Albert K., Chmielarz P., Domanskyi A. (2020). Back and to the Future: From Neurotoxin-Induced to Human Parkinson’s Disease Models. Curr. Protoc. Neurosci..

[59] Wenker S.D., Chamorro M.E., Vota D.M., Callero M.A., Vittori D.C., Nesse A.B. (2010). Differential antiapoptotic effect of erythropoietin on undifferentiated and retinoic acid-differentiated SH-SY5Y cells. J. Cell. Biochem..

[60] Turan D., Abdik H., Şahin F., Abdik E.A. (2020). Evaluation of the neuroprotective potential of caffeic acid phenethyl ester in a cellular model of Parkinson’s disease. Eur. J. Pharmacol..

[61] Ma Z., Wei X., Fontanilla C., Noelker C., Dodel R., Hampel H., Du Y. (2006). Caffeic acid phenethyl ester blocks free radical generation and 6-hydroxydopamine-induced neurotoxicity. Life Sci..

[62] Noelker C., Bacher M., Gocke P., Wei X., Klockgether T., Du Y., Dodel R. (2005). The flavanoide caffeic acid phenethyl ester blocks 6-hydroxydopamine-induced neurotoxicity. Neurosci. Lett..

[63] Silva R.B., Santos N., Martins N., Ferreira D., Barbosa F., Souza V.O., Kinoshita A., Baffa O., Del-Bel E., Santos N.A.G. (2013). Caffeic acid phenethyl ester protects against the dopaminergic neuronal loss induced by 6-hydroxydopamine in rats. Neuroscience.

