# Supplementary material for: Neuroprotective Effects of Methyl Caffeate against Hydrogen Peroxide-Induced Cell Damage: Involvement of Caspase 3 and Cathepsin D Inhibition
Source: Biomolecules. 2020 Nov 9;10(11):1530. doi: 10.3390/biom10111530 (PMC7696984; doi:10.3390/biom10111530)
Supplement: Supplementary file 1 [file biomolecules-10-01530-s001.pdf]

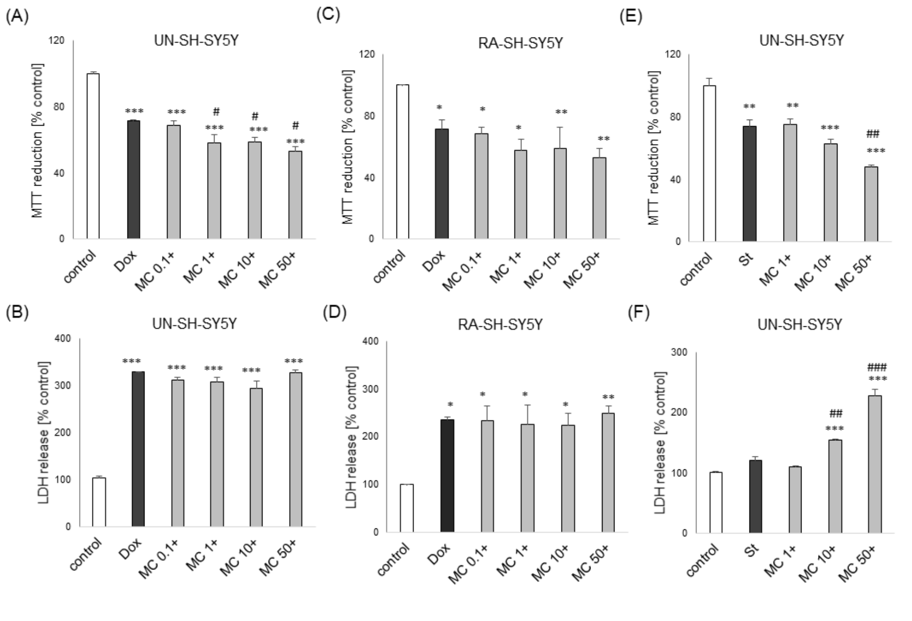

Figure S1. The effect of MC on SH-SY5Y cell damage induced by doxorubicin and staurosporine.

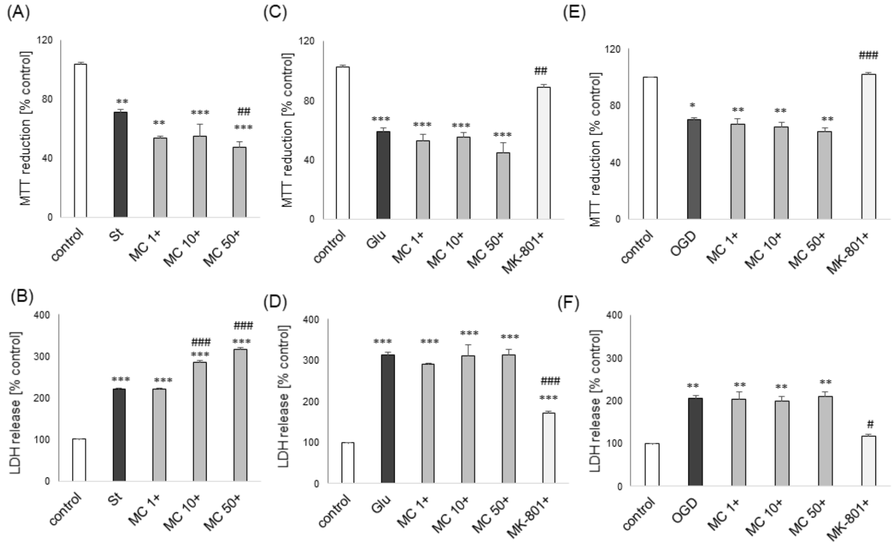

Figure S2. The effect of MC in primary neuronal cell damage induced by apoptotic (staurosporine) and excitotoxic (glutamate, oxygen glucose deprivation) factors.
